# Antimicrobial and Antibiofilm Activity against *Staphylococcus aureus* of *Opuntia ficus-indica* (L.) Mill. Cladode Polyphenolic Extracts

**DOI:** 10.3390/antiox8050117

**Published:** 2019-05-02

**Authors:** Federica Blando, Rossella Russo, Carmine Negro, Luigi De Bellis, Stefania Frassinetti

**Affiliations:** 1 Institute of Sciences of Food Production (ISPA), National Research Council (CNR), Research Unit of Lecce, Via Prov. le Lecce-Monteroni, 73100 Lecce, Italy; federica.blando@ispa.cnr.it; 2 Institute of Biology and Agricultural Biotechnology (IBBA), National Research Council (CNR), Research Unit of Pisa, Via Moruzzi 1, 56124 Pisa, Italy; rossella.wilbur@gmail.com; 3 Department of Biological and Environmental Sciences and Technologies (DiSTeBA), Salento University, 73100 Lecce, Italy; carmine.negro@unisalento.it (C.N.); luigi.debellis@unisalento.it (L.D.B.)

**Keywords:** *Opuntia ficus-indica* cladode, flavonoid, antioxidant, antimicrobial activity, antibiofilm activity

## Abstract

Plant extracts are a rich source of natural compounds with antimicrobial properties, which are able to prevent, at some extent, the growth of foodborne pathogens. The aim of this study was to investigate the potential of polyphenolic extracts from cladodes of *Opuntia ficus-indica* (L.) Mill. to inhibit the growth of some enterobacteria and the biofilm formation by *Staphylococcus aureus*. *Opuntia ficus-indica* cladodes at two stages of development were analysed for total phenolic content and antioxidant activity by Oxygen Radical Absorbance Capacity (ORAC) and Trolox equivalent antioxidant capacity (TEAC) (in vitro assays) and by cellular antioxidant activity in red blood cells (CAA-RBC) (ex vivo assay). The Liquid Chromatography Time-of-Flight Mass Spectrometry (LC/MS–TOF) analysis of the polyphenolic extracts revealed high levels of piscidic acid, eucomic acid, isorhamnetin derivatives and rutin, particularly in the immature cladode extracts. *Opuntia* cladodes extracts showed a remarkable antioxidant activity (in vitro and ex vivo), a selective inhibition of the growth of Gram-positive bacteria, and an inhibition of *Staphylococcus aureus* biofilm formation. Our results suggest and confirm that *Opuntia ficus-indica* cladode extracts could be employed as functional food, due to the high polyphenolic content and antioxidant capacity, and used as natural additive for food process control and food safety.

## 1. Introduction

In recent years, there has been an increased interest in natural antimicrobials, especially those obtained from plants. Some plant species are a rich source of natural compounds with antimicrobial properties, which are able to prevent, at some extent, the growth of foodborne pathogens, and extend the shelf life of the food [1].

Pathogens involved in foodborne diseases or food processing plant contamination are often capable to adhere and form biofilms. These structures are organized communities of bacterial cells enclosed in a self-produced polymeric matrix, composed of polysaccharides, proteins and other organic components, adhering to inert or living surfaces [2]. It is known that bacteria within biofilms are more resistant to antibiotics and other chemical agents than planktonic cells in suspension and their increased tolerance towards antimicrobial agents reduces the effectiveness to the treatment of biofilm-related infections [3,4]. Bacterial biofilms spread widely and play important roles in many industrial activities. In the dairy industry or other food processing industries or food-contact surfaces, biofilm formation is a potential source of contamination and can lead to serious hygiene problems and economic losses [4].

*Staphylococcus aureus* is a well-known pathogen living as biofilm in a wide variety of environments and represents a severe risk of food contamination. It is has been found frequently on surfaces of food processing plants and it is responsible for infections related to consumption of fresh and processed foods [5].

Phenolic compounds occurring in vegetable foods and medicinal plants have been extensively investigated against a wide range of microorganisms. Several studies demonstrated the antimicrobial activity of dietary polyphenols, their activity on bacterial growth being mainly related to the strain, the polyphenol structure, and the dosage assayed [6,7,8]. Plant extracts, rich in polyphenols, has been reported to inhibit the biofilm formation by *Staphylococcus aureus*, including methicillin-resistant *Staphylococcus aureus* MRSA, *Escherichia coli* and *Pseudomonas aeruginosa* [9,10].

*Opuntia ficus-indica* (L.) Mill. (cactus pear) is a succulent species native from America, afterwards domesticated in other countries, occupying arid and semiarid zones. The first economic importance of *Opuntia ficus-indica* relies on the production of edible fruits, consumed fresh or transformed. Cladodes (succulent stem), known as pads or nopals, are also consumed at young stage in Mexico and United States in several different food preparation, or at older stage as forage, when there is shortage of fresh forage due to droughts [11,12].

The aims of this study were to investigate the potential of polyphenolic extracts from cladodes of *Opuntia ficus-indica* to inhibit the growth of some enterobacteria and the biofilm formation by *Staphylococcus aureus*. Moreover, cladode extracts were chemically and biochemically characterized, as high performance liquid chromatography time-of-flight mass spectrometry (HPLC/MS-TOF) profile and antioxidant capacity on different in vitro and ex vivo systems.

## 2. Materials and Methods

### 2.1. Chemicals and Reagents

Reagents were purchased from various suppliers as follows: authentic standards of rutin, isoquercitrin, isorhamnetin, isorhanmetin 3-*O*-glucoside, narcissin, kaempferol (Extrasynthèse, Genay, France); FCR (Folin-Ciocalteu’s reagent), gallic acid, *p*-hydroxybenzoic acid, sodium carbonate, sodium hydroxide, 6-hydroxy-2,5,7,8-tetramethylchroman-2-carboxylic acid (Trolox), 2,2′-Azino-bis(3-ethylbenzothiazoline-6-sulfonic acid) diammonium salt (ABTS), 2,2′-Azobis (2-methylpropionamidine) dihydrochloride (AAPH), fluorescein sodium salt and 2′,7′-dichlorofluorescein diacetate (DCFH-DA), as well as acetonitrile (HPLC grade), ethanol, methanol, formic acid (Sigma-Aldrich, St. Louis, MO, USA). In all experiments, Milli-Q (Merck Millipore, Darmstadt, Germany) water was used.

### 2.2. Plant Material and Extraction

*Opuntia ficus-indica* cladodes were collected from plants producing purple fruits (‘*Rossa*’ variety), in the Salento countryside (Apulia Region, Italy, N 40°21′18″; E 17°59′47″).

Cladodes were collected from the same plants at two developmental stages: immature (from spring shoots, fully developed cladodes at 3 weeks of development) and mature (from fall shoots, old cladodes at around 24 weeks).

Cladodes were cleaned from spines and glochids, cut in small pieces, processed in a Waring blender with liquid nitrogen, freeze-dried using a Freezone^®^ 2.5 model 76530 lyophilizer (Labconco Corp., Kansas City, MO, USA) for 48 h and stored at −20 °C. This product was defined as the dry weight (DW) of cladode. Extraction was done in triplicate from 500 mg (DW) macerated with 25 mL aqueous methanol (80%) overnight at 4 °C. After centrifugation (4000× *g*), the supernatant was recovered and evaporated in vacuo at 32 °C using a R-205 Büchi rotavapor (Büchi Labortechnik AG, Essen, Germany), then re-suspended in distilled water to a concentration of 50 mg/mL. Extracts were then filtered on a 0.45-μm CA syringe filter (Filtres Fioroni, Ingré, France) and portioned in 1-mL aliquot, stored at −20 °C until analysis.

### 2.3. Identification and Quantification of Phenolic Compounds

The identification and quantification of phenolic compounds in cladode extracts was performed in triplicate (an analysis for each of the three extractions/replicas) using an Agilent 1200 Liquid Chromatography system (Agilent Technologies, Palo Alto, CA, USA) and the chromatographic conditions and column were the same already reported by Sabella et al. [13]. The identification of phenolic compounds was confirmed by a TOF LC/MS system (Agilent 6320, Agilent Technologies, Palo Alto, CA, USA), equipped with a dual ESI interface operating in negative ion mode [13].

The identified phenolic compounds were quantified by the external standard method using a six-point calibration curve of *p*-hydroxybenzoic acid (0.5–100 mg/L), rutin (0.5–50 mg/L), isorhamnetin (0.5–50 mg/L), and narcissin (0.5–50 mg/L).

### 2.4. In Vitro Antioxidant Activity

The Folin–Ciocalteu reducing capacity assay, the antioxidant activity by Oxygen Radical Absorbance Capacity (ORAC) and Trolox equivalent antioxidant capacity (TEAC) were evaluated in cladode extracts, as described in [14]. A rapid microplate methodology, using a microplate reader (Victor^TM^ X3, Perkin Elmer, Waltham, MA, USA) and 96-well plates (Costar, 96-well black round bottom plate, Corning) were used. All experiments were performed in triplicate, and two independent assays were performed for each sample.

### 2.5. Ex Vivo Antioxidant Activity

#### 2.5.1. Cellular Antioxidant Activity (CAA-RBC) Assay in Red Blood Cells

The antioxidant activity of *Opuntia* cladode extracts was evaluated in an ex vivo erythrocytes system as described in Frassinetti et al. [15] as well as erythrocytes preparation. Trolox was used as a standard and the fluorescence was read at 485 nm excitation and 535 nm emission by using a Victor^TM^ X3 microplate reader. Human erythrocytes from random subjects were exposed to a peroxyl radical generator, the AAPH, following one hour pre-treatment with 500 and 1000 µg/mL of cladode extracts. Each value was express according to the formula:

CAA unit = 100 − (∫_SA_⁄∫_CA_) × 100

where ∫_SA_ is the integrated area of the sample curve and ∫_CA_ is the integrated area of the control curve [16].

#### 2.5.2. Erythrocytes Oxidative Hemolysis

Hemolysis of human erythrocytes was induced by thermal decomposition of AAPH as previously described [15]. Briefly, erythrocytes were incubated with Trolox 500 µM (antioxidant standard) and cladode extracts at 37 °C for 1 h, followed by incubation with 50 mM AAPH at 37 °C for 4 h. The erythrocytes hemolysis was evaluated by spectrophotometric reading (λ = 540 nm) of the hemoglobin released in the supernatant, and expressed as percentage compared to the control (AAPH-treated erythrocytes).

### 2.6. Antimicrobial Activity

#### 2.6.1. Bacterial Media

Nutrient Broth (NB), Nutrient Agar (NA), Mueller Hinton Broth (MHB), Mueller Hinton Agar (MHA), Mc Farland standard 0.5, Tripticase Soy Broth (TSB), were purchased from Oxoid (Basingstone, UK).

#### 2.6.2. Bacterial Strains and Growth Conditions

The pathogenic bacterial strains were obtained from the American Type Culture Collection (ATCC). Gram-negative bacteria [*Escherichia coli* (ATCC 25922), *Salmonella enterica* ser. *Typhimurium* (ATCC 14028), and *Enterobacter aerogenes* (ATCC 13048)], and Gram-positive bacteria [*Enterococcus faecalis* (ATCC 29212) and *Staphylococcus aureus* (ATCC 25923)] were employed to test the antimicrobial activities of cladode extracts. All bacteria strains were grown on NB and MHB and incubated overnight at 37 °C under aerobic conditions.

#### 2.6.3. Inhibition Assay–MINIMUM Inhibitory Concentration (MIC)

The minimal inhibitory concentration (MIC) against selected bacteria was determined according to [7] with some modifications. Cladode extracts were diluted in sterile water to the concentration of 2000 µg/mL; then, further dilutions were made up to the concentration of 50 µg/mL.

Tested pathogenic microorganisms were cultured in MHB at 37 °C for 16 h. Initially, the cultures were diluted to match the turbidity of 0.5 McFarland standard; thereafter, further dilutions with sterile MHB made it possible to obtain a suspension of about 1–5 × 10^5^ CFU/mL. Aliquot of bacterial suspensions (50 µL) were added to a sterile 96-well plate containing 100 µL of MHB and 100 µL of cladode extract dilutions. A positive control (without cladode extract) was included on each microplate.

The plates were incubated in aerobic conditions at 37 °C for 24 h. A microplate reader (Eti-System Fast Reader Sorin Biomedica, Modena, Italy) was used to record the optical density (OD) at 600 nm. The MIC was defined as the lowest concentration of cladode extract able to inhibit the microorganism’s growth.

### 2.7. Biofilm Production and Inhibition Assay

The biofilm production was determined using the method described by [2] with some modifications. The assay was performed using two *Staphylococcus* strains: the biofilm producer *Staphylococcus aureus* (ATCC 35556) and *Staphylococcus epidermidis* (ATCC 12228), used as a negative control, since it does not produce biofilm.

The strains were activated by culturing in 5 mL of TSB at 37 °C for 24 h. After 24 h, the optical density (OD) was measured at 600 nm and appropriate dilutions were made in TSB + 1% sucrose, to obtain an optical density of 0.1 corresponding to about 10^6^ cells/mL. The assay was performed in sterile 96-well polystyrene plate (Greiner Bio-One Gmbh, Austria). Briefly, 100 µL *Staphylococcus aureus* cells were inoculated and cultured with or without 100 µL of cladodes extract (at concentrations ranging from 50 to 1500 µg/mL), without shaking at 37 °C. After 24 h incubation, non-adherent cells were removed by dipping each sample three times in sterile PBS. Samples were fixed at 60 °C for 1 h and the biofilms were stained with 0.1% solution of crystal violet in water, according to [17]. After staining, samples were washed thrice with distilled water. The quantitative analysis of biofilm production was performed by adding 125 µL of 30% acetic acid to de-stain the samples. Afterwards, the OD at 492 nm was detected using the microplate reader. The percentage of biofilm inhibition was determined by the formula:

$$Biofilm\;reduction\;\%=\frac{OD\;control\;-\;OD\;sample}{OD\;control}\times 100\%$$

### 2.8. Statistical Analysis

Statistical analysis has been carried out using GraphPad Prism, version 6.00 for Windows (GraphPad software, San Diego, CA, USA). Results were expressed as mean values ± standard deviation (SD) of analysis of data deriving from extractions and assays made in triplicate. Differences between samples were analyzed by one-way analysis of variance (ANOVA) with Bonferroni’s multiple comparison test; *p*-value lower than 0.05 was considered statistically significant.

## 3. Results and Discussion

### 3.1. Polyphenolic Composition of Opuntia ficus-indica Cladode Extracts

**Figure 1:**
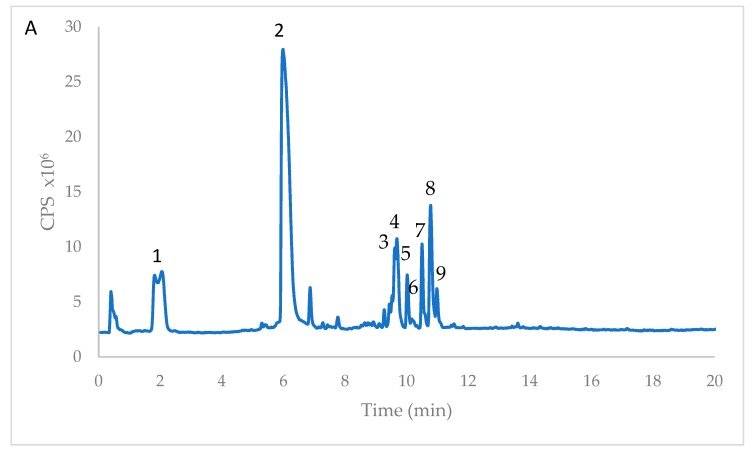
High performance liquid chromatography time-of-flight mass spectrometry (HPLC/MS-TOF) chromatogram of *Opuntia ficus-indica* extract from immature cladodes (*imm*). The chromatographic profile of mature cladode (*m*) extract was similar, except for isorhamnetin glucoside (peak 9), only present in *imm*. The numbers indicating peaks refer to the identified compounds reported in Table 1.

The polyphenolic extracts from immature and mature cladodes revealed a predominant presence of piscidic and eucomic acids. These phenolic acids were identified upon their retention time, UV-Visible spectra and fragmentation pattern; also their mass spectral characteristics (by TOF LC/MS) were compared with the available literature [18,19] (Figure 1).

The presence of piscidic acid and eucomic acid in *Opuntia* cladode has been reported [18,20]; others reported the occurrence of hydroxybenzoic acids derivatives (2- or 4- or 3,4-dihydroxy benzoic acids) [21].

In the mature cladodes, we found a higher content of both piscidic and eucomic acids in respect to the value reported (on a similar development stage) by [18], threefold and tenfold, respectively (Table 2).

In the immature cladodes, we found a much higher value, as sum of piscidic and eucomic acids, even if the ratio is inverted: in immature cladode the predominant form is eucomic acid, instead in mature cladode piscidic acid is predominant (Table 2). Anyway, the amount of piscidic and eucomic acids found in *O. ficus-indica* cladode, particularly at immature stage, is exceptionally high.

Flavonol compounds (mainly isorhamnetin derivatives and rutin) were also identified (Figure 1 and Table 1). Isorhamnetin derivatives and rutin have been already described to be present in cladode extracts of different *Opuntia* species [18,19,20,21,22,23]. In those papers, isorhamnetin was reported as predominant core aglycone for flavonoids present in *Opuntia* sp., with low occurrence of quercetin and kaempferol. We confirmed these findings (Figure 1 and Table 2).

The content of isorhamnetin glucoside in our immature sample was similar to that one reported by Guevara-Figueroa and co-workers [21], in a wild variety (Amarillo) of *Opuntia ficus-indica*, instead our reported content of narcissin was much higher, as well as the rutin content (Table 2). In a mature cladode sample, the content of isorhamnetin derivatives (totaling 1.76 mg/g DW) was similar to that one reported in the above study (1.69 mg/g DW) [21]. The total flavonols content in our immature samples (6.78 mg/g DW) was in agreement with the content (8.82 mg/g DW) reported in young cladodes of *Opuntia ficus-indica* [23]. In mature sample, the total flavonols content (totalling 2.51 mg/g DW) was again in agreement with [21] who found ~3.5 mg /g DW.

Isorhamnetin derivatives, piscidic and eucomic acids present in such a high amount in cladode of *Opuntia ficus-indica* indicate that nopal can be considered a promising plant for the development of polyphenol-based commercial products. The effect of these phenolic compounds has been evaluated in vitro for contrasting hypercholesterolemia [24], and against UVA-induced oxidative stress on human keratinocytes [25], suggesting a possible pharmaceutical use of cladode extracts.

### 3.2. In Vitro Antioxidant Activity

Cladode extracts analysis revealed a much higher total phenol content, as well as antioxidant capacity, at immature stage of development, than mature cladode (Table 3). Therefore, the habit of Mexican population to eat ‘nopalitos’ (that is young prickly pads of 3–4 weeks of age) relays on a scientific basis of higher health benefit.

Total phenol content (Table 3) was higher than that one reported in some studies [21,26] but in agreement with [23] (for immature cladode), and similar to that one reported in mature cactus pads from Tenerife [27] (for mature cladode); in fact, the phenol level results higher in immature cladodes if compared with mature, e.g., about 4 g/kg in our immature cladode samples from Apulia, and 2.6 g/kg in mature cladodes as reported by Rocchetti et al. for Sicilian *Opuntia ficus-indica* [26].

The antioxidant capacity measured by ABTS assay resulted higher for immature cladode than mature (12.5 vs 8.2 µmol TE/g DW, respectively) (Table 3). Only one study dealt with TEAC of cladode extracts reporting a several-fold higher value [28]. The ORAC values for our immature and mature cladode (Table 3) agreed with the literature: ~770 µmol TE/g DW [28]; a range from 260–380 µmol TE/g DW, in young pads [19]; 657 µmol TE/g DW [29]. The ORAC value for immature cladode extracts was comparable to the value reported for blueberry; instead, that one for mature cladode was comparable to strawberry/raspberry values [30].

Therefore, from the experiments assessing the antioxidant capacity of cladodes extracts, we can conclude that both immature and mature cladodes are a good source of antioxidant functional compounds.

### 3.3. Ex Vivo Antioxidant Activity

#### 3.3.1. Cellular Antioxidant Activity Assay in Red Blood Cells (CAA-RBC)

**Figure 2:**
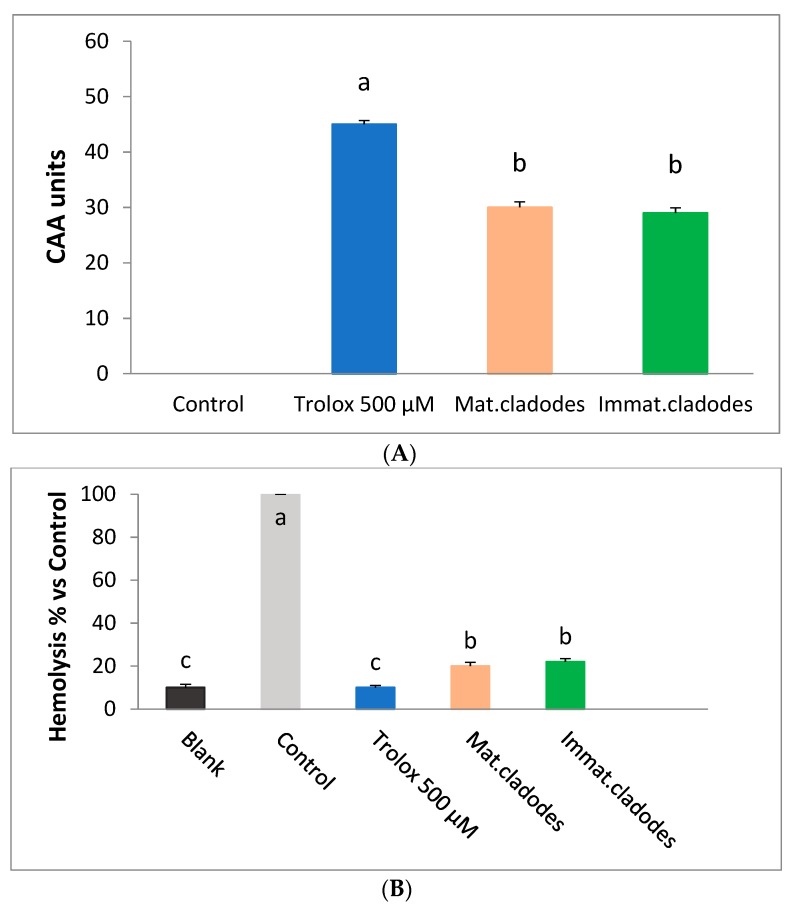
(**A**) Effects of *Opuntia ficus-indica* cladode extracts on the cellular antioxidant activity (CAA) in human erythrocytes. Trolox was used as reference standard. (**B**) Effects of *Opuntia ficus-indica* cladodes extracts on 2,2′-Azobis (2-methylpropionamidine) dihydrochloride (AAPH)-induced hemolysis in human erythrocytes. Trolox was used as reference standard. Assays were carried out in triplicate and the results were expressed as mean values ± SD. Different letters indicate significant differences (*p* ≤ 0.05). One-way ANOVA with Bonferroni’s multiple comparison test.

As shown in Figure 2A, cladodes pre-treated erythrocytes exhibited a significantly higher cellular antioxidant activity (CAA unit = 30 ± 1) compared to untreated cells (control, CAA = 0; *p* < 0.01), but lower than Trolox (CAA unit = 45 ± 0.79) used as standard. The cellular antioxidant activity of mature and immature cladodes was similar for both samples (30 ± 1 and 29 ± 0.9, respectively).

#### 3.3.2. Haemolysis Assay

Since erythrocyte is a unique cell model with simple metabolism and sensitivity to oxidation, oxidative haemolysis is widely used to evaluate antioxidant activity. Therefore, cladode extracts were tested on human erythrocytes to counteract the oxidative hemolysis induced by peroxyl radicals produced by AAPH thermal decomposition. The anti-hemolytic activity of cladode extracts was compared with that from erythrocytes exposed to AAPH alone (control). As shown in Figure 2B both *Opuntia* cladodes extracts exhibited a strong anti-hemolytic effect (about 80%). This effect was statistically significant (*p* ≤ 0.001) compared to the control (that induced 100% of hemolysis) but lower than the Trolox (about 90% hemolysis inhibition). No statistically significant differences were found between mature and immature cladodes anti-hemolytic effects.

### 3.4. Antimicrobial Activity

The antimicrobial activity against selected enteric bacterial strains was measured evaluating the O.D. at 600 nm in the presence of increasing doses of cladode extract. Different dilutions of methanol did not affect the bacterial growth (data not shown).

Gram-negative microorganisms (*Escherichia coli* ATCC 25922, *Salmonella typhimurium* ATCC 14028 and *Enterobacter aerogenes* ATCC 13048) were inhibited at a concentration of 2000 µg/mL of mature cladode extract, whereas immature cladode extract was more effective inhibiting at a concentration of 1500 µg/mL (Table 4). The two Gram-positive bacteria (*Staphylococcus. aureus* ATCC 25923 and *Enterococcus. faecalis* ATCC 29212) showed MIC values of 1500 µg/mL for mature and 1000 for immature cladode extract (Table 4). The MIC against planktonic cells of the biofilm producer *Staphylococcus aureus* ATCC 35556 was 1000 and 700 µg/mL for mature and immature extracts, respectively (Table 4).

Our results agree with several other studies showing that the inhibitory effect of phenolic compounds from natural extracts are more effective to Gram-positive than Gram-negative bacteria [7]. The susceptibility of bacteria to drugs depends on the characteristics of the drug (hydrophobicity or hydrosolubility) and on the microbial membrane composition [31].

The antimicrobial activity of plant phenolics has been extensively investigated against many different microorganisms [6]. We previously reported the antimicrobial activity of bergamot whole-fruit powder against potentially pathogenic bacteria [32]. In general, phenolic compounds are involved in membrane damage, protein and cell wall binding and enzyme inactivation; they can act as pro-oxidants, leading to damage of DNA, lipids and other biological molecules [33].

Few authors have reported the antimicrobial activity of *Opuntia ficus-indica*. Ginestra and co-workers [18] reported that different phytochemical fractions of *Opuntia ficus-indica* did not show antimicrobial activity against the tested bacterial strains. On the other hand, it has been reported the antimicrobial activity of alcoholic and aqueous extracts of *Opuntia* cladodes against *Vibrio cholerae* and *Proteus mirabilis* [34]. Moreover, other authors [35] described the antimicrobial activity of *Opuntia* cladodes against *Escherichia coli* and *Staphylococcus aureus*, with a minimum bactericidal concentration (MBC) of 4 mg/mL and 1 mg/mL, respectively.

The antimicrobial activity of *Opuntia* cladodes extracts may be related to its high content of polyphenols, especially isorhamnetin that has been already reported exerting antimicrobial activity [36].

### 3.5. Biofilm Inhibition

The effects of cladode extracts on biofilm formation by *Staphylococcus aureus* ATCC 35556, a strong biofilm producer, was also investigated.

**Figure 3:**
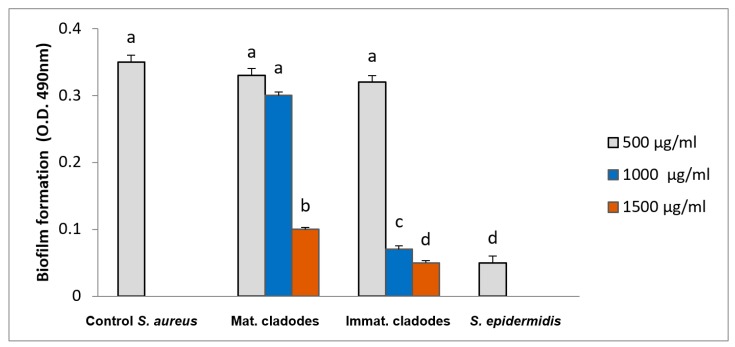
Effects of different concentrations of *Opuntia ficus-indica* cladode extracts on biofilm formation by *Staphylococcus aureus* ATCC 35556. *Staphylococcus epidermidis* ATCC 12228 was used as negative control. Assays were carried out in triplicate and the results were expressed as mean values ± SD. Different letters indicate significant differences (*p* ≤ 0.05). One-way ANOVA with Bonferroni’s multiple comparison test.

As shown in Figure 3, both cladode extracts (mature and immature) at concentration of 500 µg/mL did not inhibited the biofilm formation. At 1000 µg/mL, only the immature cladode extract inhibited significantly the biofilm formation with an inhibition rate of 80%. At 1500 µg/mL concentration, both extracts significantly inhibited the biofilm formation, with an inhibition rate of 71% for the mature cladode and 85% for the immature cladode.

The inhibition of biofilm development at concentration of *Opuntia* cladode extracts higher than MIC demonstrated that the bacterial cells in a biofilm are more resistant to antimicrobial agents compared to the planktonic cells, which is a well-known feature. Our results concerning the inhibition of *Staphylococcus aureus* MRSA biofilm formation agree with previous works [10,37]. In our study, the inhibition of biofilm could be explained by the presence of flavonoids in cladodes extracts. In fact, flavonoids such as quercetin, apigenin, luteolin and rutin were found to be effective in the inhibition of *Staphylococcus aureus* biofilm [38,39].

## 4. Conclusions

The results obtained in this work indicate that cladodes of *Opuntia ficus-indica* are rich in phenolic compounds, particularly *p*-hydroxybenzoic acid derivatives, and possess in vitro antioxidant activity which results higher in the immature cladodes. The extracts exhibited in vivo antioxidant capacity with a strong anti-hemolytic effect. A selective inhibition against potentially pathogenic bacteria and a significant inhibition of the biofilm formation were shown by the cladodes at both developmental stages. To our knowledge, this is the first report on the characterization of *Opuntia ficus-indica* cladodes extracts for their antimicrobial and anti-biofilm properties. In conclusion, our results indicate that *Opuntia ficus-indica* cladode extracts, especially the immature ones, could be used for food purpose as functional food (being comparable to the antioxidant value of blueberries), as well as for food process control and food safety as recently proposed by Rocchetti et al. [26].

## Figures and Tables

**Table 1:** Identification of phenolic acids and flavonols in *Opuntia ficus-indica* extracts (from *imm* and *m*) by means of High performance liquid chromatography time-of-flight mass spectrometry HPLC/TOF (M − H)^−^.

Peak	RT	Formula [M − H]^−^	m/z Exp	m/z Calc	Δ (ppm)	Compound Name	Reference
**1**	1.988	C_11_H_11_O_7_	255.0512	255.0510	−0.67	Piscidic acid	Ginestra et al., 2009 [18]
**2**	6.170	C_11_H_11_O_6_	239.0567	239.0561	−2.52	Eucomic acid	Ginestra et al., 2009 [18]
**3**	9.628	C_34_H_41_O_20_	769.6770	769.6773	−0.39	Isorhamnetin rhamnosyl rutinoside	Santos-Zea et al., 2011 [19]
**4**	9.695	C_33_H_39_O_20_	755.6512	755.6508	0.53	Isorhamnetin glucosyl rhamnosyl pentoside	Santos-Zea et al., 2011 [19]
**5**	10.022	C_27_H_29_O_16_	609.1459	609.1461	0.34	Rutin	Ginestra et al., 2009 [18]
**6**	10.034	C_27_H_29_O_16_	609,1470	609,1461	−1.43	Isorhamnetin sambubioside	Santos-Zea et al., 2011 [19]
**7**	10.503	C_27_H_29_O_15_	593.1517	593.1512	−0.88	Kampherol glucosyl rhamnoside	Santos-Zea et al., 2011 [19]
**8**	10.785	C_28_H_31_O_16_	623.1645	623.1618	−4.37	Narcissin (isorhamnetin rutinoside)	Santos-Zea et al., 2011 [19]
**9**	10.987	C_22_H_21_O_12_	477.1045	477.1038	−1.42	Isorhamnetin glucoside	Ginestra et al., 2009 [18]

**Table 2:** Phenolic acids and flavonols content of *Opuntia ficus-indica* extracts, from immature and mature cladodes. Phenolic acids were quantified using the calibration curve of *p*-hydroxybenzoic acid. Flavonols were determined using calibration curves with the closest appropriate standard (rutin, isorhamnetin glucoside or isorhamnetin rutinoside). Mean values (*n* = 3) ± standard deviation (SD) are expressed as dry weight (DW) or fresh weight (FW). The same letters (*a–h*) in the column indicate that mean values are not significantly different (*p* < 0.05).

**Immature Stage**	**mg/g DW**	**mg/100g FW**
(1) Piscidic acid	1.984 ± 0.019 *c*	14.681 ± 0.146 *c*
(2) Eucomic acid	13.506 ± 0.143 *a*	99.944 ± 1.062 *a*
(4) Isorhamnetin rhamnosyl rutinoside	0.411 ± 0.003 *d*	3.041 ± 0.027 *e*
(5) Isorhamnetin glucosyl rhamnosyl pentoside	0.296 ± 0.004 *e*	2.192 ± 0.033 *d*
(6) Rutin	2.030 ± 0.023 *c*	15.022 ± 0.171 *c*
(8) Narcissin (isorhamnetin rutinoside)	3.188 ± 0.042 *b*	23.598 ± 0.302 *b*
(9) Isorhamnetin glucoside	0.465 ± 0.021 *d*	3.444 ± 0.161 *e*
**Mature Stage**	**mg/g DW**	**mg/100g FW**
(1) Piscidic acid	3.281 ± 0.032 *b*	28.257 ± 0.279 *b*
(2) Eucomic acid	1.616 ± 0.02 *f*	13.917 ± 0.174 *f*
(4) Isorhamnetin rhamnosyl rutinoside	0.187 ± 0.012 *e*	1.618 ± 0.104 *d*
(5) Isorhamnetin glucosyl rhamnosyl pentoside	0.266 ± 0.007 *e*	2.293 ± 0.067 *d*
(6) Rutin	0.752 ± 0.004 *g*	6.474 ± 0.034 *g*
(8) Narcissin (isorhamnetin rutinoside)	1.160 ± 0.029 *f*	9.985 ± 0.25 *h*
(9) Isorhamnetin glucoside	ND	ND

**Table 3:** Total phenols and antioxidant capacity, expressed as Oxygen Radical Absorbance Capacity (ORAC) and Trolox equivalent antioxidant capacity (TEAC), of *Opuntia ficus-indica* extracts, from immature and mature cladodes. Total phenols are expressed as gallic acid equivalents (GAE), TEAC and ORAC assays are expressed as Trolox Equivalent (TE). Mean values (*n* = 11) ± standard deviation (SD) are expressed on a dry weight (DW) or fresh weight (FW) basis. The same letters (a,b) in the same column indicate that mean values are not significantly different (*p* < 0.05).

	Total Phenols		TEAC		ORAC	
	mg GAE/g DW	mg GAE/100 g FW	μmol TE/g DW	μmol TE/100 g FW	mmol TE/g DW	mmol TE/100 g FW
*Immature stage*	54.02 ± 1.62 *a*	399.61 ± 11.98 *a*	12.55 ± 1.14 *a*	92.87 ± 8.51 *a*	0.88 ± 0.08 *a*	6.52 ± 0.56 *a*
*Mature stage*	18.63 ± 0.94 *b*	160.14 ± 8.09 *b*	8.23 ± 0.72 *b*	70.85 ± 6.22 *b*	0.29 ± 0.04 *b*	2.47 ± 0.36 *b*

**Table 4:** Minimal inhibitory concentrations (MIC) of *Opuntia ficus-indica* mature (*m*) and immature cladode extracts (*imm*) against selected bacterial strains.

Strain		MIC (*m*) µg/mL	MIC (*imm*) µg/mL
*Escherichia coli*	ATCC 25922	2000	1500
*Salmonella typhimurium*	ATCC 14028	2000	1500
*Enterobacter aerogenes*	ATCC 13048	2000	1500
*Enterococcus faecalis*	ATCC 29212	1500	1000
*Staphylococcus aureus*	ATCC 25923	1500	1000
*Staphylococcus aureus*	ATCC 35556	1000	700
